# Retinal nerve fiber layer thickness in multiple sclerosis with and without optic neuritis: a four-year follow-up study from Oman

**DOI:** 10.1186/s12886-021-02158-0

**Published:** 2021-11-12

**Authors:** Abdullah S. Al-Mujaini, Maiysa S. Al-Mujaini, Buthaina I. Sabt

**Affiliations:** 1 Department of Ophthalmology, College of Medicine and Health Sciences, Sultan Qaboos University, Muscat, Oman; 2 College of Medicine and Health Sciences, Sultan Qaboos University, Muscat, Oman; 3 Department of Ophthalmology, Sultan Qaboos University Hospital, Muscat, Oman

**Keywords:** Optic neuritis, Multiple sclerosis, Retinal nerve Fiber layer, Optical coherence tomography

## Abstract

**Background:**

Multiple sclerosis (MS) is an autoimmune disease that attacks the central nervous system, with optic neuritis (ON) being a common early manifestation. Retinal nerve fiber layer (RNFL) thickness may be a biomarker of neuroaxonal damage in MS patients. We sought to evaluate changes in RNFL thickness over 4 years in Omani MS patients with or without ON in comparison to a healthy control group.

**Methods:**

This retrospective case-control study involved 27 MS patients and 25 healthy controls. Optical coherence tomography was performed upon first diagnosis and at a four-year follow-up. Differences in mean RNFL thickness were calculated.

**Results:**

A total of 51 eyes from the MS group and 50 eyes from the control group were evaluated. There was a significant reduction in mean RNFL thickness among MS patients with ON at follow-up (81.21 versus 72.14 μm; *P* = .003), whereas no significant RNFL thinning was observed among MS patients without ON. However, there was a significant reduction in RNFL thickness among MS patients compared to healthy controls (76.79 versus 93.72 μm; *P* = .009), regardless of ON presence/absence.

**Conclusions:**

Axonal damage was seen in the optic nerves of Omani MS patients. Moreover, there was a significant reduction in RNFL thickness among MS patients with ON as the disease progressed; however, while there was evidence of RNFL thinning in MS patients without ON, this difference lacked statistical significance. Evaluation of RNFL thickness may represent a useful biomarker for monitoring disease progression in MS and its association with ON.

## Background

Multiple sclerosis (MS) is a chronic autoimmune disorder, which attacks the central nervous system (CNS) and damages the myelin sheath, oligodendrocytes and nerve fibers [1, 2]. It is considered a neurodegenerative and inflammatory disease of the CNS [1]. A common initial manifestation of MS is optic neuritis (ON), a type of nerve inflammation secondary to demyelination which damages the nerve fibers and myelin sheath of the optic nerve, leading to impaired vision or blindness [3].

Recently, reduced thickness of the retinal nerve fiber layer (RNFL) has been proposed as a possible biomarker of disability and neurodegeneration in MS patients [4, 5]. While it may be difficult to accurately discern the impact of MS on the CNS, the RNFL is more readily accessible for the purposes of clinical examination since it is made up of densely-packed non-myelinated axons [6]. In particular, optical coherence tomography (OCT) is a diagnostic tool which can be used to assess RNFL thickness and thus monitor disease progression in both ON and MS [7–9]. This study therefore aimed to compare RNFL thickness using OCT among Omani MS patients with or without ON over a four-year period in comparison with a healthy control group.

## Methods

This retrospective case-control study was conducted between January 2011 and December 2015 at the ophthalmology and neurology clinics of the Sultan Qaboos University Hospital (SQUH), Muscat, Oman. The target population included Omani patients who had been referred to the ophthalmology clinic by a specialized neurologist following a confirmed diagnosis of MS based on the 2010 McDonald criteria [10]. Non-Omani patients, those with other ocular diseases such as retinal vessel diseases, glaucoma, neuromyelitis optica, and uveitis and those with other CNS disorders were excluded from the study.

All patients underwent complete ophthalmic examinations and OCT imaging to determine the presence of ON. Subsequently, the study group was divided into two arms, comprising MS patients with ON (*n* = 14) and those without ON (*n* = 13). In addition, healthy individuals (*n* = 25) were recruited to form a control group for comparative purposes. Data were collected from the subjects’ electronic medical records, including their age, gender, medical history, OCT imaging findings, date of first and last OCT imaging, the presence or absence of ON in each eye, and RNFL thickness in both eyes. All subjects were followed up for a period of 4 years.

During the OCT examination, a peripapillary ring scan was performed using the SPECTRALIS® SD-OCT system (Heidelberg Engineering Inc.) to measure RNFL thickness. All OCT scans were performed by a single experienced ophthalmic technician using a signal strength of > 6 and an internal fixation target. A pupil diameter of at least 4 mm was required for scanning. No manual correction was applied to the OCT output. Imaging of the retina was performed cross-sectionally and all parameters were measured automatically. Measurements of RNFL thickness were calculated using a circular papillary map. The quality of the scans was assessed and poor-quality scans were rejected prior to the analysis of data. Patients with ON were recruited and underwent OCT imaging 6 months after their initial ON episode.

Collected data were analyzed using the Statistical Package for the Social Sciences (SPSS), version 23 (IBM Corp., Armonk, NY). Continuous variables were assessed for normal distribution. For demographic data, descriptive statistics were calculated using frequency tables and differences were assessed using a Chi-squared test. Changes in RNFL thickness between baseline and follow-up OCT imaging were determined using a Wilcoxon signed-rank test. A one-way analysis of variance (ANOVA) was conducted to compare differences in OCT findings and other continuous variables between groups. Significant results from the ANOVA test were subsequently assessed using a post-*hoc* test. The level of statistical significance was established at *P* < .05.

Ethical approval for this study was obtained from the Medical Research & Ethics Committee of the College of Medicine & Health Sciences at Sultan Qaboos University (MREC approval #1950). Further authorization was obtained from the hospital information system to access patients’ medical records. Informed consent was obtained from all patients. All methods were performed in accordance with the relevant guidelines and regulations adhered to the tenets of the Declaration of Helsinki as amended in 2008.

## Results

A total of 51 eyes from 27 MS patients and 50 eyes from 25 healthy controls were evaluated. The mean age was 30 and 34 years old among MS patients and healthy controls, respectively. The MS group comprised seven males (25.9%) and 20 females (74.1%), while the control group comprised nine males (36%) and 16 females (64%). Among the 27 MS patients, 14 (51.9%) were diagnosed with ON, of which three (21.4%) had unilateral ON, resulting in 25 affected eyes. Eye involvement was sequential in all patients with bilateral ON. The remaining 13 patients (48.1%) with MS had no history of ON in either eye (Table 1).

**Table 1:** Characteristics of Omani patients with multiple sclerosis in comparison to a control group (*N* = 52)

Participants (n)	Control group	MS group		
		Total	With ON	Without ON
Subjects	25	27	14	13
Eyes	50	51^a^	25^a^	26
**Age (years)**				
Mean	34	30	28	34
**Gender (n [%])**				
Male	9 (36)	7 (25.9)	4 (28.6)	3 (23.1)
Female	16 (64)	20 (74.1)	10 (71.4)	10 (76.9)
**Visual acuity**	–	0.718	0.728	0.709
**MS subtype (n [%])**				
**RR**	–	25 (92.6)	13 (92.9)	12 (92.3)
**SP**	–	1 (3.7)	0 (0)	1 (7.7)
**CIS**	–	1 (3.7)	1 (7.1)	0 (0)

Abbreviations: *MS* multiple sclerosis, *ON* optic neuritis, *RR* relapsing-remitting, *SP* secondary progressive, *CIS* clinically isolated syndrome

^a^Including three MS patients with unilateral ON

**Fig. 1:**
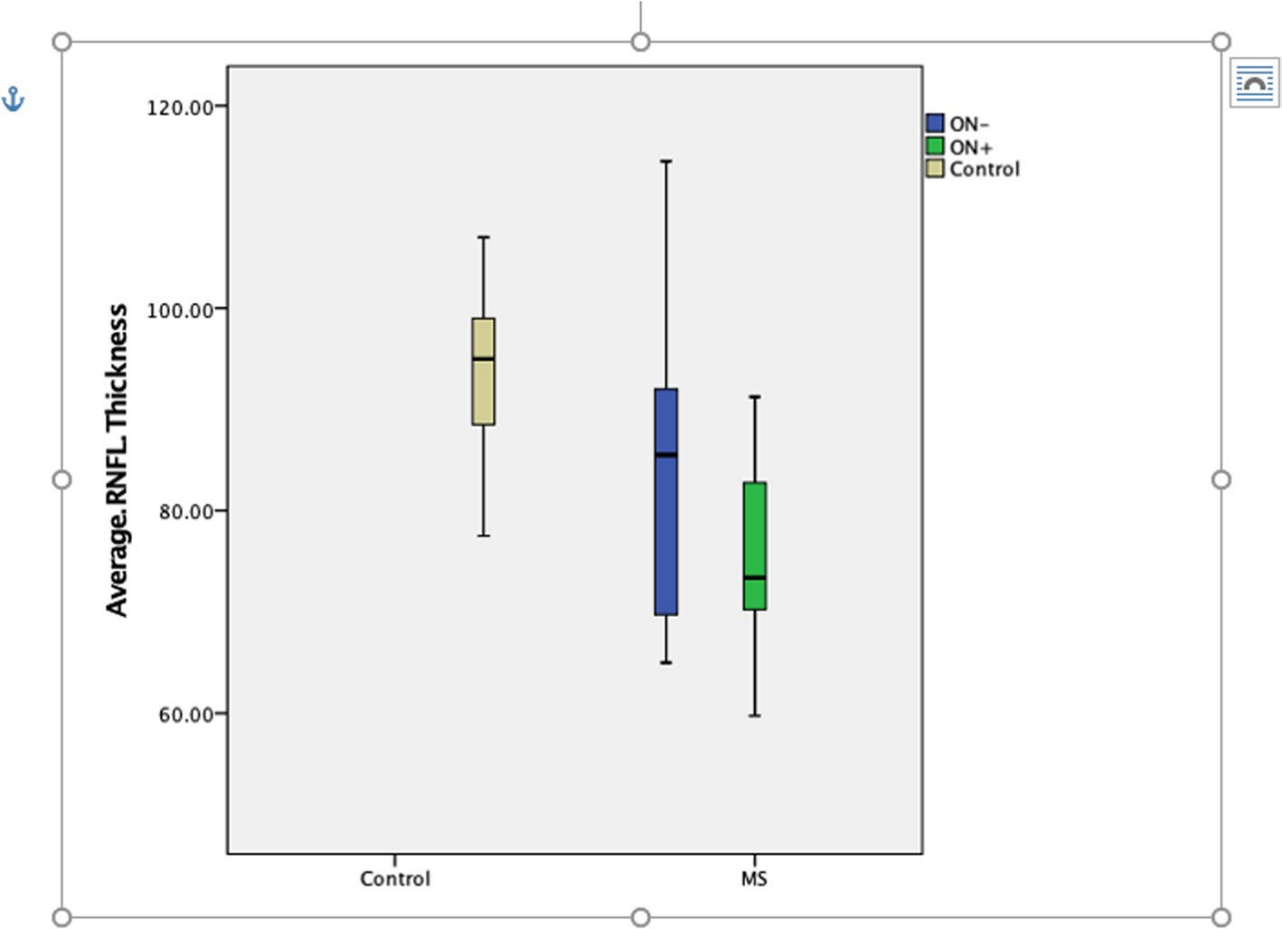
Retinal nerve fiber layer (RNFL) thickness over time among Omani multiple sclerosis (MS) patients with and without optic neuritis (ON) in comparison to a control group (*N* = 52)

Among MS patients with ON, there was a significant loss of average RNFL thickness in the affected eyes at the four-year follow-up evaluation in comparison to OCT measurements from baseline (81.21 versus 72.14 μm; *P* = .003). Among MS patients without ON, there was a slight albeit non-significant thinning in RNFL thickness over the same period (85.35 versus 81.81 μm; *P* = .223). Nevertheless, while ON-affected eyes showed greater RNFL thinning compared to non-affected eyes, the difference in mean RNFL thickness between the two groups at follow-up was not statistically significant (72.14 versus 81.81 μm; *P* = .081) (Fig. 1).

**Fig. 2:**
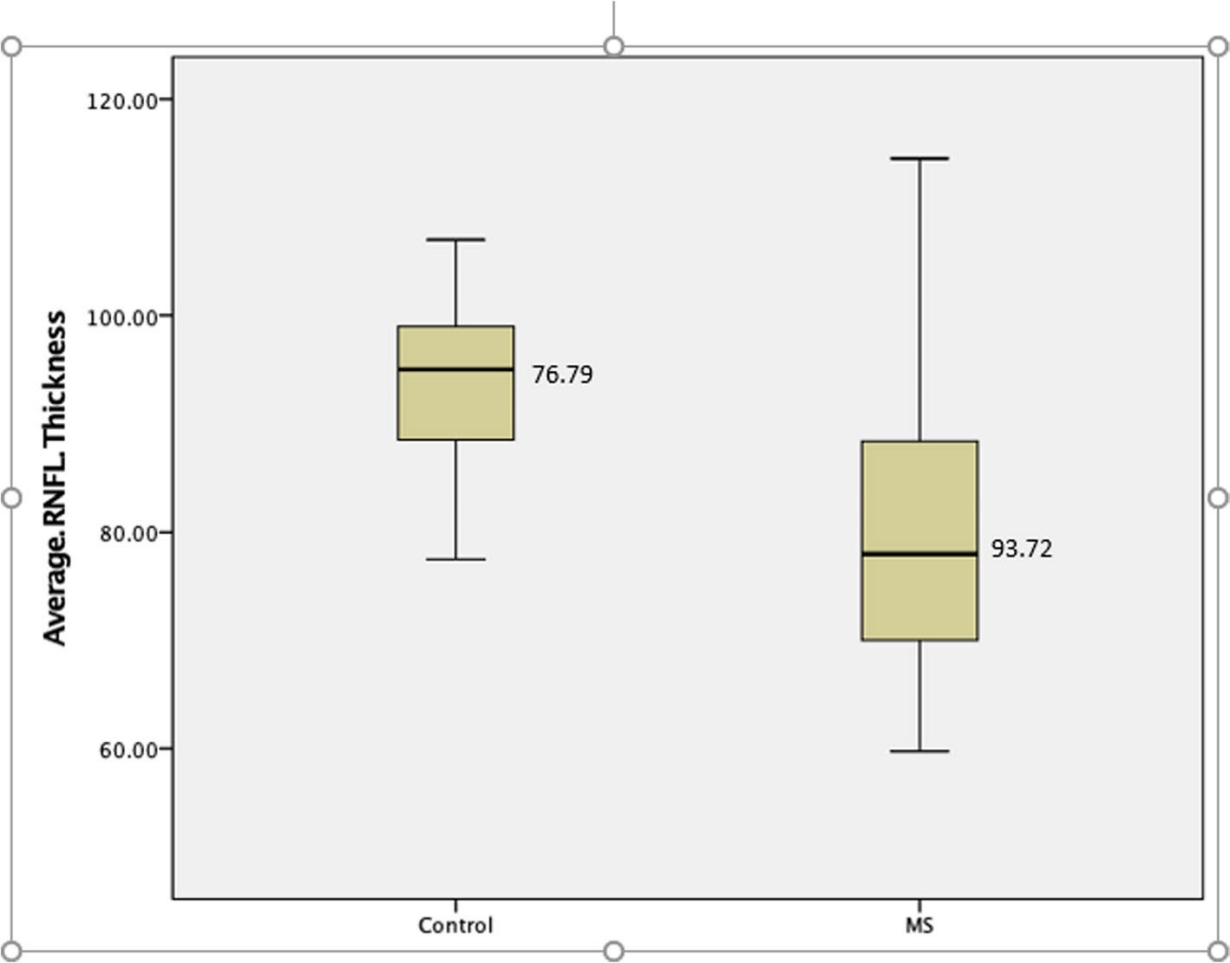
Retinal nerve fiber layer (RNFL) thickness among Omani multiple sclerosis patients in comparison to a control group (*N* = 52)

Compared to those of the control group, the eyes of MS patients showed a highly significant reduction in RNFL thickness over time for both patients with and without ON (*P =* .002 and .010, respectively) (Fig. 1). Moreover, an examination of the eyes of all MS patients, regardless of the absence or presence of ON, revealed significant RNFL thinning in comparison to the healthy controls (76.79 versus 93.72 μm; *P* = .009) (Fig. 2).

## Discussion

Worldwide, the prevalence of MS varies widely within different populations. According to a hospital-based study, the prevalence of MS in Oman from 2006 to 2019 was 15.9 cases per 100,000 individuals [11]. This rate is extremely low compared to much higher rates reported in North America and Europe (over 100 cases per 100,000 individuals), as well as elsewhere in the Arabian Gulf region (31–55 cases per 100,000 individuals), thus suggesting that the country is a low or medium-risk zone for MS [11–13]. Nevertheless, previous data indicates that the prevalence of MS in Oman from 1990 to 2000 was only four cases per 100,000 individuals [14]. Thus, the rate of MS in Oman seems to mirror global trends in showing an increase in prevalence over time. In addition, the clinical profile of Omani MS patients appears to be very similar to that of other populations reported in Asia, in that a high proportion present with visual and motor symptoms and approximately one-third are diagnosed with opticospinal disease [14].

The main goal of the current study was to determine whether RNFL loss occurs in the presence or absence of ON in MS patients. This was achieved by detecting changes in RNFL thickness among Omani MS patients both with and without ON over a period of 4 years in comparison to healthy controls. Our findings indicated that there was a significant reduction in RNFL thickness in the affected eyes of MS patients with ON when comparing baseline and final OCT imaging measurements over a prolonged period of time.

These results are consistent with those from previous reports. Costello et al. [15] observed that 74% of MS patients demonstrated RNFL thinning within 3–6 months of an ON diagnosis, with a significant reduction in RNFL thickness in ON-affected compared to non-affected eyes. A prior study by Garcia-Martin et al [7] concluded that MS patients with ON experienced a significant reduction in RNFL thickness over a period of 5 years. Balk et al [16] similarly reported that the loss of RNFL thickness in eyes with MS-associated ON occurs over time (mean thickness at baseline: 78 μm, absolute change over time: − 1.1 μm, 95% confidence interval: 1.4–0.7 μm; *P* < .001). According to Feng et al [6], disease duration was inversely related to average RNFL thickness among MS patients. In contrast, Henderson et al [9] reported that there was no relationship between disease duration and RNFL thinning in ON-affected eyes. Likewise, both Eyre et al [17] and Balk et al [16] found no significant relationship between number of ON episodes or relapses and mean RNFL thickness.

In the present study, a comparison of baseline and follow-up OCT measurements indicated that changes in RNFL thickness over time among MS patients without ON were not statistically significant. Similarly, a study performed by Henderson et al [9] found a non-significant association between mean RNFL thickness and disease duration in selected eyes with no known history of ON. Khanifar et al [18] stated that RNFL thinning was correlated with an increased risk of ON, and that such thinning may represent a predictive cutoff point for the presence or absence of ON. However, other studies have reported conflicting findings. A study conducted by Gelfand et al [19] in the United States demonstrated that RNFL reduction begins in the early stages of MS, independently of ON. Similarly, Balk et al [20] found that the eyes of MS patients without a history of ON also demonstrated a significant reduction in RNFL thickness. In a two-year follow-up study, Garcia-Martin et al [21] found that RNFL changes occurred at a similar rate in non-affected eyes with no evidence of ON. Eslami et al [22] found a significant inverse correlation between MS duration and RNFL thickness, regardless of the presence or absence of ON.

No significant difference in RNFL thickness were observed between ON-affected and non-affected eyes at a four-year follow-up in the current study. However, there was a significantly greater loss of RNFL thickness in ON-affected eyes compared to non-affected eyes. In line with these findings, Gelfand et al [19] reported that even though eyes affected by ON demonstrated greater RNFL thinning compared to those without ON, the difference between the two groups was not statistically significant. Another study also revealed non-significant differences in RNFL thinning between affected and unaffected eyes of unilateral ON patients (80.72 ± 18.18 μm versus 99.53 ± 13.26 μm; *P* = .149) [23]. These assumptions are also supported by findings from other research [24, 25]. Nevertheless, a study by Feng et al [6] conducted in China concluded that there was a significant difference in mean RNFL thickness between the eyes of MS patients with and those without a history of ON (71.8 ± 19.2 μm versus 92.0 ± 8.5 μm; *P* = .001). Khanifar et al [18] also reported similar results (83.0 versus 90.5 μm; *P* = .02). Other studies have also found reduction in RNFL thickness to be significantly greater in eyes with a history of ON compared to unaffected eyes and the eyes of healthy controls [26, 27].

In our study, we demonstrated a significant reduction in mean RNFL thickness among MS patients compared to healthy controls. In line with these results, a study performed in Germany by Bock et al [8] indicated that average RNFL loss was significantly greater among MS patients compared to healthy controls. Studies from China (81.9 ± 17.8 μm versus 102.1 ± 8.1 μm; *P* = .00) and the USA (88.5 versus 97 μm; *P* < .001) confirmed significant RNFL thinning in the MS group compared to the control group [6, 18]. Saxena et al [23] also reported a significant difference in RNFL thickness in the nasal (66.23 ± 12.4 μm versus 88.93 ± 22.18 μm; *P* < .001) and superior (106.77 ± 17.92 μm versus 132.33 ± 15.42 μm; *P* < .001) quadrants of the eyes of MS patients when compared to the healthy group. These findings can be explained by the fact that the optic nerve has a high density of axons, which makes it vulnerable to atrophy. However, Garcia-Martin et al [7] reported non-significant differences in RNFL changes over a prolonged follow-up period of 5 years when comparing the eyes of MS patients with those of a healthy control group.

Finally, there was significant RNFL thinning in the eyes of MS patients both with and without ON in the present study when compared separately with the control group. These significant correlations can be understood in light of previous studies. A study from Spain noted progressive thinning over 5 years in the eyes of MS patients both with and without a history of ON when compared to healthy controls (change over time: − 3.5 μm, − 4.7 μm, and − 2.2 μm, respectively) [28]. Garcia-Martin et al [7] also found that MS patients exhibited a greater reduction in RNFL thickness than healthy controls, regardless of ON history. However, a prior study conducted in the United Kingdom found that there was no significant difference in RNFL thickness between MS eyes with no history of ON compared to a healthy control group [26].

The current study provides valuable information on OCT changes in Omani MS patients. However, the main limitation was the small sample size, a factor which the authors attribute to the rarity of the disease in the Omani population. This limitation of low sample size due to the rarity of MS in Oman might be the reason that no significant reduction of RNFL over time was identified in MS patients without ON, even though there was a reduction from 85.35 to 81.81 μm. Moreover, due to the retrospective design of the study and the reliance on chart review as the primary method of data collection, it is possible that some data might be missing which may have resulted in selective bias. Finally, the majority of patients in the study had bilateral ON, a relatively rare finding in MS patients; unfortunately, testing of myelin oligodendrocyte glycoprotein antibodies could not be performed as this facility has only been implemented at SQUH within the last 3 years. Further studies are therefore recommended to address these limitations. In addition, the authors recommend the assessment of ganglion cell-inner plexiform layer thickness as another line of research into potential biomarkers of MS severity and disease progression.

## Conclusion

MS appears to result in progressive changes in the RNFL thickness over time irrespective of the occurrence of ON. In the presence of ON, a significant reduction in RNFL thickness was noted among Omani MS patients as the disease progressed with time. In contrast, MS patients with no history of ON demonstrated non-significant RNFL thinning over the same period. Regardless of the presence or absence of ON, there was a significant difference in RNFL thickness between MS patients and healthy controls. As such, measurement of RNFL thickness may represent a useful biomarker for evaluating progression of the disease and its association with ON.

## Data Availability

The datasets used and/or analyzed during the current study are available from the corresponding author on reasonable request.
